# Crystal structure of phenyl *N*-(4-nitro­phen­yl)carbamate

**DOI:** 10.1107/S2056989015021544

**Published:** 2015-11-21

**Authors:** Y. AaminaNaaz, Subramaniyan Sathiyaraj, Sundararaj Kalaimani, A. Sultan Nasar, A. SubbiahPandi

**Affiliations:** a Department of Physics, Presidency College (Autonomous), Chennai 600 005, India; b Department of Polymer Science, University of Madras, Guindy Campus, Chennai 600 025, India

**Keywords:** crystal structure, carbamate, ester, nitro­phenyl carbamate, hydrogen bonding, C—H⋯π inter­action

## Abstract

The asymmetric unit of the title compound, C_13_H_10_N_2_O_4_, contains two independent mol­ecules (*A* and *B*). The dihedral angle between the aromatic rings is 48.18 (14)° in mol­ecule *A* and 45.81 (14)° in mol­ecule *B*. The mean plane of the carbamate N—C(=O)—O group is twisted slightly from the attached benzene and phenyl rings, making respective dihedral angles of 12.97 (13) and 60.93 (14)° in *A*, and 23.11 (14) and 59.10 (14)° in *B*. In the crystal, *A* and *B* mol­ecules are arranged alternately through N—H⋯O hydrogen bonds and C—H⋯π inter­actions, forming chains along the *a* axis. The chains are further linked by C—H⋯O hydrogen bonds into a double-chain structure.

## Related literature   

For the details of biological activity of carbamate derivatives, see: O’Donnell *et al.* (1979 ▸); Bubert *et al.* (2007 ▸). For applications of the carbamate group as a building block in crystal engineering, see: Ghosh *et al.* (2006 ▸). For polymorphs of phenyl carbamate, see: Wishkerman & Bernstein (2008 ▸).
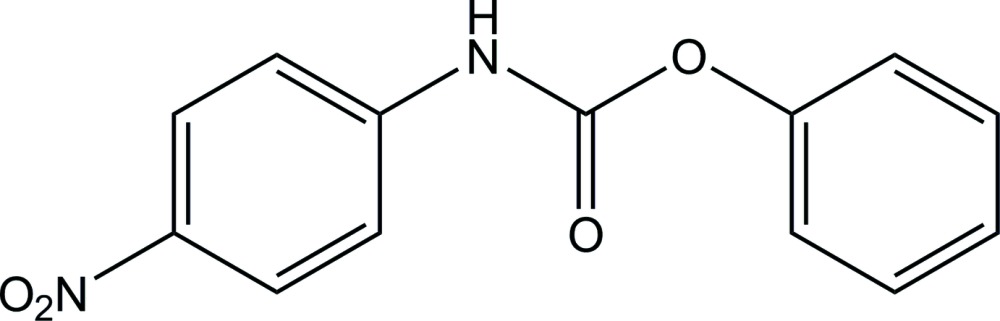


## Experimental   

### Crystal data  

C_13_H_10_N_2_O_4_
*M* *_r_* = 258.23
Triclinic, 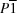

*a* = 9.6722 (3) Å
*b* = 10.2543 (5) Å
*c* = 12.4787 (6) Å
α = 84.625 (3)°
β = 79.386 (3)°
γ = 77.955 (3)°
*V* = 1187.73 (9) Å^3^
*Z* = 4
Mo *K*α radiation
μ = 0.11 mm^−1^
*T* = 293 K
0.20 × 0.18 × 0.17 mm

### Data collection  

Bruker Kappa APEXII CCD diffractometer
Absorption correction: multi-scan (*SADABS*; Bruker, 2004 ▸) *T*_min_ = 0.978, *T*_max_ = 0.982
26582 measured reflections
4755 independent reflections
2713 reflections with *I* > 2σ(*I*)
*R*_int_ = 0.051

### Refinement  

*R*[*F* ^2^ > 2σ(*F* ^2^)] = 0.054
*wR*(*F* ^2^) = 0.127
*S* = 1.05
4755 reflections
343 parameters
H-atom parameters constrained
Δρ_max_ = 0.20 e Å^−3^
Δρ_min_ = −0.18 e Å^−3^

### 

Data collection: *APEX2* (Bruker, 2004 ▸); cell refinement: *SAINT* (Bruker, 2004 ▸); data reduction: *SAINT*; program(s) used to solve structure: *SHELXS97* (Sheldrick, 2008 ▸); program(s) used to refine structure: *SHELXL2014* (Sheldrick, 2015 ▸); molecular graphics: *ORTEP-3 for Windows* (Farrugia, 2012 ▸); software used to prepare material for publication: *PLATON* (Spek, 2009 ▸).

## Supplementary Material

Crystal structure: contains datablock(s) global, I. DOI: 10.1107/S2056989015021544/is5423sup1.cif

Structure factors: contains datablock(s) I. DOI: 10.1107/S2056989015021544/is5423Isup2.hkl

Supporting information file. DOI: 10.1107/S2056989015021544/is5423Isup3.cml

**Fig. 1.**
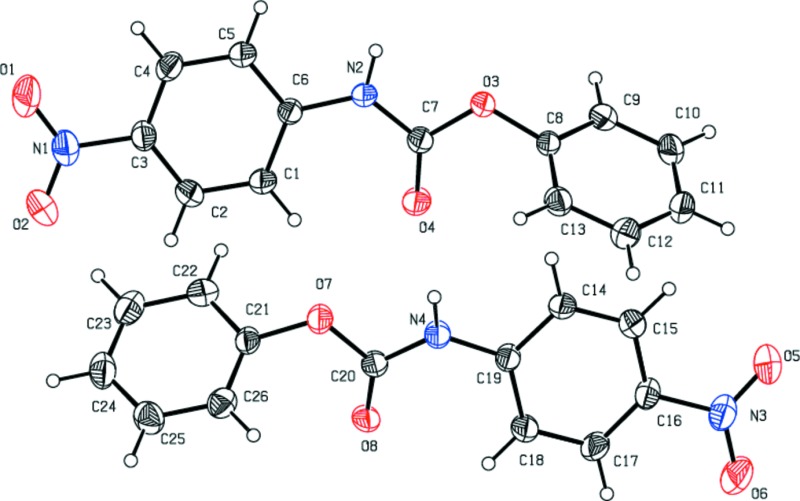
A view of two independent molecules (A and B) of the title compound, showing the atom labelling. Displacement ellipsoids are drawn at the 30% probability level.

A B . DOI: 10.1107/S2056989015021544/is5423fig1.tif
A view of two independent mol­ecules (*A* and *B*) of the title compound, showing the atom labelling. Displacement ellipsoids are drawn at the 30% probability level.

**Fig. 2.**
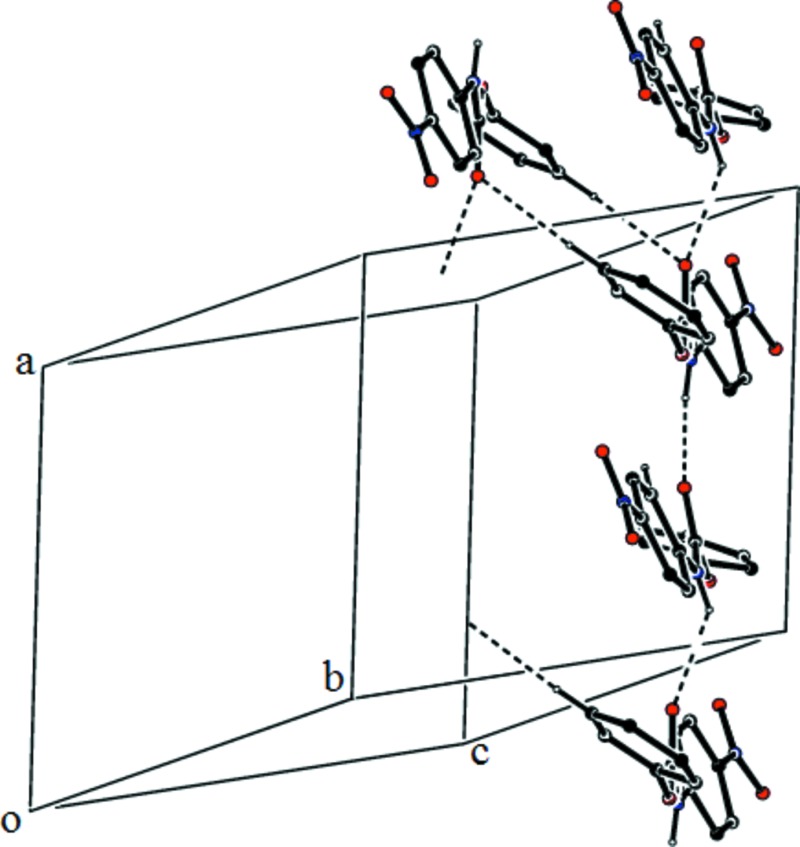
A partial packing view of the title compound. The N—H···O and C—H···O hydrogen bonds are indicated by dashed lines.

. DOI: 10.1107/S2056989015021544/is5423fig2.tif
A partial packing view of the title compound. The N—H⋯O and C—H⋯O hydrogen bonds are indicated by dashed lines.

CCDC reference: 1436927

Additional supporting information:  crystallographic information; 3D view; checkCIF report

## Figures and Tables

**Table 1:** Hydrogen-bond geometry (Å, °) *Cg*2 is the centroid of the C8–C13 ring.

*D*—H⋯*A*	*D*—H	H⋯*A*	*D*⋯*A*	*D*—H⋯*A*
N2—H2⋯O8^i^	0.86	2.35	3.057 (2)	140
N4—H4⋯O4	0.86	2.05	2.906 (2)	171
C25—H25⋯O8^ii^	0.93	2.57	3.448 (4)	158
C14—H14⋯*Cg*2	0.93	2.94	3.592 (2)	128
C17—H17⋯*Cg*2^iii^	0.93	2.94	3.736 (3)	144

Symmetry codes: (i) 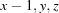; (ii) 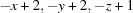; (iii) 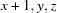.

## supplementary crystallographic information

### S1. Comment

The carbamate group is known in biochemistry for its role in biological process. For example it tunes haemoglobin affinity for O_2_ during physiological respiration (O'Donnell *et al.*, 1979). Carbamate derivatives present significant pharmacological activity, in some cases exhibiting potential anticancer drugs (Bubert *et al.*, 2007). In the solid state, the carbamate group acts as both donor and acceptor in hydrogen bonding, favoring the formation of highly stable synthons. Thus, the carbamate group has been proposed in crystal engineering as a building block for hydrogen bonded solids (Ghosh *et al.*, 2006). Most carbamate compounds of interest are phenyl derivatives. In the known polymorphs of one such compound, phenyl carbamate, the molecular environment is very similar around the carbamate group but very different around the phenyl ring (Wishkerman & Bernstein, 2008). As part of our studies in this area, we report herein on the synthesis of phenyl 4-nitrophenylcarbamate.

Two molecules (*A* and *B*) present in the asymmetric unit (Fig. 1) have nitrophenyl groups exhibiting planar conformations, with their maximum deviations of 0.0207 Å for atom O1 in molecule *A* and 0.0186 Å for atom N3 in molecule *B*. In molecule *A* the nitrophenyl ring (C1–C6) makes a dihedral angle of 48.18 (14)° with the phenyl ring (C8–C13), while in *B* the C14–C19 ring makes a dihedral angle of 45.81 (41)° with the C21–C26 ring. In the crystal, molecules are linked to one another by N—H···O and C—H···π interactions (Table 1), leading to formation of a hydrogen bonded chain along [100] (Fig. 2). A C—H···O interaction is also observed between the chains.

### S2. Experimental

To a stirred solution of 1.0 g (5.45 mmol) of 4-nitroaniline dissolved in 100 ml of dry THF was added calculated amount with 5% excess of phenylchloroforamate in 50ml of dry THF. The addition rate was such that it took 1.5 hrs for complete transfer. After the addition was over, the stirring was continued overnight. Excess THF was removed under vacuum at room temperature. The crude product was extracted with ethyl acetate (3×100 ml). The organic layer was dried over anhydrous sodium sulphate. Removal of solvent under vacuum at room temperature yielded light yellow product and the product was dried under vacuum to constant weight. Light yellow crystals were obtained by slow evaporation of an ethyl acetate solution at room temperature (yield 99%).

### S3. Refinement

The N- and C-bound H atoms were positioned geometrically (N—H = 0.86 Å and C—H = 0.93–0.97 Å) and allowed to ride on their parent atoms, with *U*_iso_(H) = 1.2*U*_eq_(N, C).

### Crystal data

**Table:**

C_13_H_10_N_2_O_4_	*Z* = 4
*M**_r_* = 258.23	*F*(000) = 536
Triclinic, *P*1	*D*_x_ = 1.444 Mg m^−^^3^
*a* = 9.6722 (3) Å	Mo *K*α radiation, λ = 0.71073 Å
*b* = 10.2543 (5) Å	Cell parameters from 2713 reflections
*c* = 12.4787 (6) Å	θ = 1.7–26.7°
α = 84.625 (3)°	µ = 0.11 mm^−^^1^
β = 79.386 (3)°	*T* = 293 K
γ = 77.955 (3)°	Block, yellow
*V* = 1187.73 (9) Å^3^	0.20 × 0.18 × 0.17 mm

### Data collection

**Table:**

Bruker Kappa APEXII CCD diffractometer	2713 reflections with *I* > 2σ(*I*)
Radiation source: fine-focus sealed tube	*R*_int_ = 0.051
ω and φ scan	θ_max_ = 26.7°, θ_min_ = 1.7°
Absorption correction: multi-scan (*SADABS*; Bruker, 2004)	*h* = −12→12
*T*_min_ = 0.978, *T*_max_ = 0.982	*k* = −12→12
26582 measured reflections	*l* = −15→15
4755 independent reflections	

### Refinement

**Table:**

Refinement on *F*^2^	Primary atom site location: structure-invariant direct methods
Least-squares matrix: full	Secondary atom site location: difference Fourier map
*R*[*F*^2^ > 2σ(*F*^2^)] = 0.054	Hydrogen site location: inferred from neighbouring sites
*wR*(*F*^2^) = 0.127	H-atom parameters constrained
*S* = 1.05	*w* = 1/[σ^2^(*F*_o_^2^) + (0.0314*P*)^2^ + 0.7359*P*] where *P* = (*F*_o_^2^ + 2*F*_c_^2^)/3
4755 reflections	(Δ/σ)_max_ = 0.001
343 parameters	Δρ_max_ = 0.20 e Å^−^^3^
0 restraints	Δρ_min_ = −0.18 e Å^−^^3^

### Special details

**Table:**

Geometry. All e.s.d.'s (except the e.s.d. in the dihedral angle between two l.s. planes) are estimated using the full covariance matrix. The cell e.s.d.'s are taken into account individually in the estimation of e.s.d.'s in distances, angles and torsion angles; correlations between e.s.d.'s in cell parameters are only used when they are defined by crystal symmetry. An approximate (isotropic) treatment of cell e.s.d.'s is used for estimating e.s.d.'s involving l.s. planes.

### Fractional atomic coordinates and isotropic or equivalent isotropic displacement parameters (Å^2^)

**Table:**

	*x*	*y*	*z*	*U*_iso_*/*U*_eq_	
O1	0.1987 (3)	1.4653 (3)	0.2962 (2)	0.1211 (11)	
O2	0.4178 (3)	1.3760 (3)	0.2866 (2)	0.1112 (10)	
O3	0.14804 (16)	0.94691 (18)	0.85885 (14)	0.0536 (5)	
O4	0.36223 (17)	0.98269 (19)	0.76845 (16)	0.0623 (6)	
O5	0.6977 (2)	0.6060 (2)	1.24825 (17)	0.0808 (7)	
O6	0.9181 (3)	0.5613 (3)	1.1749 (2)	0.1056 (9)	
O7	0.64969 (17)	1.0924 (2)	0.67313 (15)	0.0636 (6)	
O8	0.86028 (16)	1.01542 (18)	0.73187 (14)	0.0503 (5)	
N1	0.2949 (3)	1.3872 (3)	0.3303 (2)	0.0700 (7)	
N2	0.15095 (19)	1.0939 (2)	0.72024 (16)	0.0459 (6)	
H2	0.0601	1.1063	0.7436	0.055*	
N3	0.7953 (3)	0.6162 (2)	1.1734 (2)	0.0624 (7)	
N4	0.6566 (2)	0.9385 (2)	0.80607 (17)	0.0538 (6)	
H4	0.5708	0.9423	0.7947	0.065*	
C1	0.3347 (2)	1.1489 (3)	0.5694 (2)	0.0488 (7)	
H1	0.4065	1.0882	0.5980	0.059*	
C2	0.3674 (3)	1.2201 (3)	0.4728 (2)	0.0525 (7)	
H2A	0.4612	1.2078	0.4353	0.063*	
C3	0.2609 (3)	1.3093 (3)	0.4319 (2)	0.0471 (7)	
C4	0.1224 (3)	1.3294 (3)	0.4854 (2)	0.0528 (7)	
H4A	0.0513	1.3904	0.4565	0.063*	
C5	0.0896 (3)	1.2587 (3)	0.5818 (2)	0.0496 (7)	
H5	−0.0044	1.2722	0.6189	0.060*	
C6	0.1948 (2)	1.1675 (2)	0.62459 (19)	0.0391 (6)	
C7	0.2340 (3)	1.0057 (3)	0.7803 (2)	0.0439 (6)	
C8	0.2096 (2)	0.8366 (3)	0.9213 (2)	0.0413 (6)	
C9	0.1760 (3)	0.8419 (3)	1.0324 (2)	0.0519 (7)	
H9	0.1232	0.9194	1.0641	0.062*	
C10	0.2220 (3)	0.7299 (3)	1.0962 (2)	0.0597 (8)	
H10	0.1989	0.7312	1.1719	0.072*	
C11	0.3018 (3)	0.6165 (3)	1.0489 (2)	0.0613 (8)	
H11	0.3318	0.5408	1.0922	0.074*	
C12	0.3366 (3)	0.6156 (3)	0.9380 (2)	0.0588 (8)	
H12	0.3931	0.5395	0.9061	0.071*	
C13	0.2898 (3)	0.7250 (3)	0.8724 (2)	0.0499 (7)	
H13	0.3121	0.7233	0.7968	0.060*	
C14	0.5899 (2)	0.8313 (3)	0.9790 (2)	0.0491 (7)	
H14	0.4949	0.8680	0.9737	0.059*	
C15	0.6218 (3)	0.7541 (3)	1.0701 (2)	0.0509 (7)	
H15	0.5492	0.7392	1.1273	0.061*	
C16	0.7617 (3)	0.6991 (3)	1.0757 (2)	0.0472 (7)	
C17	0.8703 (3)	0.7182 (3)	0.9922 (2)	0.0574 (8)	
H17	0.9647	0.6782	0.9970	0.069*	
C18	0.8386 (3)	0.7970 (3)	0.9014 (2)	0.0586 (8)	
H18	0.9117	0.8111	0.8444	0.070*	
C19	0.6979 (2)	0.8553 (3)	0.8949 (2)	0.0438 (6)	
C20	0.7362 (3)	1.0130 (3)	0.7368 (2)	0.0459 (7)	
C21	0.7079 (2)	1.1696 (3)	0.5852 (2)	0.0446 (7)	
C22	0.6464 (3)	1.3018 (3)	0.5788 (2)	0.0579 (8)	
H22	0.5761	1.3390	0.6350	0.070*	
C23	0.6904 (3)	1.3792 (3)	0.4876 (3)	0.0634 (8)	
H23	0.6487	1.4693	0.4817	0.076*	
C24	0.7946 (3)	1.3247 (3)	0.4059 (2)	0.0636 (8)	
H24	0.8240	1.3774	0.3445	0.076*	
C25	0.8553 (3)	1.1925 (3)	0.4147 (2)	0.0611 (8)	
H25	0.9269	1.1554	0.3593	0.073*	
C26	0.8121 (3)	1.1135 (3)	0.5045 (2)	0.0523 (7)	
H26	0.8533	1.0232	0.5101	0.063*	

### Atomic displacement parameters (Å^2^)

**Table:**

	*U*^11^	*U*^22^	*U*^33^	*U*^12^	*U*^13^	*U*^23^
O1	0.0914 (18)	0.159 (3)	0.0944 (19)	−0.0135 (17)	−0.0231 (15)	0.0760 (19)
O2	0.0793 (17)	0.134 (2)	0.0881 (18)	−0.0056 (15)	0.0246 (14)	0.0447 (16)
O3	0.0328 (9)	0.0660 (13)	0.0531 (11)	−0.0047 (8)	−0.0035 (8)	0.0224 (10)
O4	0.0300 (10)	0.0772 (14)	0.0786 (14)	−0.0164 (9)	−0.0170 (8)	0.0295 (11)
O5	0.0936 (16)	0.0871 (17)	0.0543 (13)	−0.0163 (13)	−0.0073 (12)	0.0208 (12)
O6	0.0705 (16)	0.129 (2)	0.108 (2)	−0.0078 (14)	−0.0356 (14)	0.0576 (17)
O7	0.0348 (9)	0.0821 (14)	0.0709 (13)	−0.0169 (9)	−0.0156 (9)	0.0368 (11)
O8	0.0310 (10)	0.0694 (13)	0.0513 (11)	−0.0153 (8)	−0.0097 (7)	0.0104 (9)
N1	0.0719 (18)	0.081 (2)	0.0531 (16)	−0.0175 (15)	−0.0082 (14)	0.0196 (14)
N2	0.0286 (10)	0.0535 (14)	0.0510 (13)	−0.0052 (9)	−0.0061 (9)	0.0132 (11)
N3	0.0702 (17)	0.0595 (17)	0.0613 (17)	−0.0180 (13)	−0.0243 (14)	0.0153 (13)
N4	0.0299 (11)	0.0725 (16)	0.0599 (14)	−0.0180 (10)	−0.0155 (10)	0.0251 (12)
C1	0.0329 (13)	0.0548 (17)	0.0563 (17)	−0.0070 (11)	−0.0096 (11)	0.0102 (14)
C2	0.0391 (14)	0.0621 (19)	0.0530 (17)	−0.0124 (13)	−0.0011 (12)	0.0062 (15)
C3	0.0491 (15)	0.0500 (17)	0.0435 (15)	−0.0159 (12)	−0.0096 (12)	0.0084 (13)
C4	0.0469 (15)	0.0531 (17)	0.0579 (18)	−0.0072 (12)	−0.0186 (13)	0.0139 (14)
C5	0.0351 (13)	0.0547 (17)	0.0549 (17)	−0.0056 (12)	−0.0071 (12)	0.0097 (14)
C6	0.0344 (13)	0.0403 (15)	0.0443 (14)	−0.0118 (10)	−0.0099 (10)	0.0051 (12)
C7	0.0365 (15)	0.0479 (16)	0.0472 (16)	−0.0110 (11)	−0.0080 (11)	0.0063 (13)
C8	0.0290 (12)	0.0490 (16)	0.0443 (15)	−0.0095 (11)	−0.0080 (10)	0.0126 (13)
C9	0.0414 (14)	0.0629 (19)	0.0455 (16)	−0.0051 (12)	−0.0014 (12)	0.0031 (14)
C10	0.0542 (17)	0.079 (2)	0.0406 (16)	−0.0099 (16)	−0.0061 (13)	0.0144 (16)
C11	0.0563 (17)	0.058 (2)	0.064 (2)	−0.0100 (15)	−0.0107 (14)	0.0228 (16)
C12	0.0589 (17)	0.0484 (18)	0.065 (2)	−0.0080 (13)	−0.0074 (14)	0.0034 (15)
C13	0.0479 (15)	0.0591 (19)	0.0429 (15)	−0.0130 (13)	−0.0076 (12)	0.0018 (14)
C14	0.0332 (13)	0.0536 (17)	0.0589 (17)	−0.0096 (11)	−0.0090 (12)	0.0099 (14)
C15	0.0448 (15)	0.0521 (17)	0.0533 (17)	−0.0117 (12)	−0.0043 (12)	0.0071 (14)
C16	0.0521 (16)	0.0434 (16)	0.0503 (16)	−0.0153 (12)	−0.0187 (12)	0.0105 (13)
C17	0.0390 (14)	0.0611 (19)	0.072 (2)	−0.0110 (13)	−0.0189 (13)	0.0191 (16)
C18	0.0343 (14)	0.075 (2)	0.0627 (18)	−0.0133 (13)	−0.0083 (12)	0.0220 (16)
C19	0.0365 (13)	0.0477 (16)	0.0500 (16)	−0.0154 (11)	−0.0136 (11)	0.0111 (13)
C20	0.0339 (14)	0.0576 (18)	0.0459 (15)	−0.0101 (12)	−0.0106 (11)	0.0087 (13)
C21	0.0347 (13)	0.0532 (17)	0.0479 (16)	−0.0141 (12)	−0.0143 (11)	0.0143 (13)
C22	0.0448 (15)	0.063 (2)	0.0610 (19)	−0.0023 (14)	−0.0077 (13)	0.0014 (16)
C23	0.0658 (19)	0.0501 (19)	0.078 (2)	−0.0123 (15)	−0.0277 (17)	0.0120 (17)
C24	0.0667 (19)	0.074 (2)	0.0555 (19)	−0.0270 (17)	−0.0215 (16)	0.0217 (17)
C25	0.0630 (18)	0.078 (2)	0.0417 (17)	−0.0143 (16)	−0.0068 (13)	−0.0018 (16)
C26	0.0522 (16)	0.0511 (17)	0.0557 (18)	−0.0097 (13)	−0.0163 (13)	0.0006 (14)

### Geometric parameters (Å, º)

**Table:**

O3—C7	1.347 (3)	C5—C4	1.365 (3)
O3—C8	1.402 (3)	C5—H5	0.9300
O8—C20	1.196 (3)	C14—C15	1.368 (3)
O7—C20	1.354 (3)	C14—H14	0.9300
O7—C21	1.394 (3)	C9—C10	1.377 (4)
N2—C7	1.348 (3)	C9—H9	0.9300
N2—C6	1.397 (3)	C15—C16	1.365 (3)
N2—H2	0.8600	C15—H15	0.9300
O4—C7	1.197 (3)	C13—C12	1.373 (4)
C6—C5	1.380 (3)	C13—H13	0.9300
C6—C1	1.384 (3)	C10—C11	1.372 (4)
N4—C20	1.341 (3)	C10—H10	0.9300
N4—C19	1.401 (3)	C4—C3	1.365 (3)
N4—H4	0.8600	C4—H4A	0.9300
C19—C14	1.381 (3)	C17—C16	1.368 (4)
C19—C18	1.381 (3)	C17—C18	1.373 (3)
N1—O2	1.200 (3)	C17—H17	0.9300
N1—O1	1.208 (3)	C26—C25	1.373 (4)
N1—C3	1.457 (3)	C26—H26	0.9300
C1—C2	1.370 (3)	C25—C24	1.363 (4)
C1—H1	0.9300	C25—H25	0.9300
C8—C13	1.367 (3)	C18—H18	0.9300
C8—C9	1.367 (3)	C22—C23	1.379 (4)
C21—C26	1.361 (4)	C22—H22	0.9300
C21—C22	1.363 (4)	C12—C11	1.364 (4)
O5—N3	1.213 (3)	C12—H12	0.9300
C2—C3	1.368 (3)	C11—H11	0.9300
C2—H2A	0.9300	C23—C24	1.364 (4)
N3—O6	1.206 (3)	C23—H23	0.9300
N3—C16	1.467 (3)	C24—H24	0.9300
			
C7—O3—C8	118.89 (17)	C10—C9—H9	120.7
C20—O7—C21	120.14 (18)	C16—C15—C14	119.0 (2)
C7—N2—C6	127.80 (19)	C16—C15—H15	120.5
C7—N2—H2	116.1	C14—C15—H15	120.5
C6—N2—H2	116.1	C8—C13—C12	118.3 (3)
C5—C6—C1	119.4 (2)	C8—C13—H13	120.9
C5—C6—N2	117.0 (2)	C12—C13—H13	120.9
C1—C6—N2	123.6 (2)	C11—C10—C9	120.4 (3)
C20—N4—C19	127.1 (2)	C11—C10—H10	119.8
C20—N4—H4	116.4	C9—C10—H10	119.8
C19—N4—H4	116.4	C3—C4—C5	119.2 (2)
C14—C19—C18	119.6 (2)	C3—C4—H4A	120.4
C14—C19—N4	116.9 (2)	C5—C4—H4A	120.4
C18—C19—N4	123.4 (2)	C16—C17—C18	119.4 (2)
O8—C20—N4	127.9 (2)	C16—C17—H17	120.3
O8—C20—O7	124.1 (2)	C18—C17—H17	120.3
N4—C20—O7	108.0 (2)	C15—C16—C17	121.7 (2)
O4—C7—O3	124.7 (2)	C15—C16—N3	118.8 (2)
O4—C7—N2	127.0 (2)	C17—C16—N3	119.5 (2)
O3—C7—N2	108.37 (19)	C4—C3—C2	121.5 (2)
O2—N1—O1	122.7 (3)	C4—C3—N1	118.7 (2)
O2—N1—C3	118.9 (3)	C2—C3—N1	119.9 (2)
O1—N1—C3	118.4 (3)	C21—C26—C25	118.8 (3)
C2—C1—C6	119.9 (2)	C21—C26—H26	120.6
C2—C1—H1	120.0	C25—C26—H26	120.6
C6—C1—H1	120.0	C24—C25—C26	120.7 (3)
C13—C8—C9	122.0 (2)	C24—C25—H25	119.6
C13—C8—O3	120.8 (2)	C26—C25—H25	119.6
C9—C8—O3	117.0 (2)	C17—C18—C19	119.8 (2)
C26—C21—C22	121.6 (2)	C17—C18—H18	120.1
C26—C21—O7	121.5 (2)	C19—C18—H18	120.1
C22—C21—O7	116.7 (2)	C21—C22—C23	118.7 (3)
C3—C2—C1	119.4 (2)	C21—C22—H22	120.6
C3—C2—H2A	120.3	C23—C22—H22	120.6
C1—C2—H2A	120.3	C11—C12—C13	121.2 (3)
O6—N3—O5	123.6 (3)	C11—C12—H12	119.4
O6—N3—C16	118.3 (3)	C13—C12—H12	119.4
O5—N3—C16	118.1 (2)	C12—C11—C10	119.5 (3)
C4—C5—C6	120.6 (2)	C12—C11—H11	120.2
C4—C5—H5	119.7	C10—C11—H11	120.2
C6—C5—H5	119.7	C24—C23—C22	120.6 (3)
C15—C14—C19	120.5 (2)	C24—C23—H23	119.7
C15—C14—H14	119.7	C22—C23—H23	119.7
C19—C14—H14	119.7	C25—C24—C23	119.6 (3)
C8—C9—C10	118.6 (3)	C25—C24—H24	120.2
C8—C9—H9	120.7	C23—C24—H24	120.2
			
C7—N2—C6—C5	−175.7 (3)	C14—C15—C16—C17	−0.7 (4)
C7—N2—C6—C1	7.5 (4)	C14—C15—C16—N3	−180.0 (3)
C20—N4—C19—C14	154.4 (3)	C18—C17—C16—C15	1.4 (5)
C20—N4—C19—C18	−25.8 (5)	C18—C17—C16—N3	−179.3 (3)
C19—N4—C20—O8	6.2 (5)	O6—N3—C16—C15	175.5 (3)
C19—N4—C20—O7	−171.3 (3)	O5—N3—C16—C15	−4.1 (4)
C21—O7—C20—O8	9.8 (4)	O6—N3—C16—C17	−3.8 (4)
C21—O7—C20—N4	−172.6 (2)	O5—N3—C16—C17	176.6 (3)
C8—O3—C7—O4	−10.3 (4)	C5—C4—C3—C2	−0.1 (4)
C8—O3—C7—N2	170.2 (2)	C5—C4—C3—N1	−178.8 (3)
C6—N2—C7—O4	7.1 (5)	C1—C2—C3—C4	0.1 (4)
C6—N2—C7—O3	−173.4 (2)	C1—C2—C3—N1	178.9 (3)
C5—C6—C1—C2	−0.5 (4)	O2—N1—C3—C4	175.4 (3)
N2—C6—C1—C2	176.3 (3)	O1—N1—C3—C4	−1.0 (5)
C7—O3—C8—C13	−57.6 (3)	O2—N1—C3—C2	−3.4 (5)
C7—O3—C8—C9	127.5 (3)	O1—N1—C3—C2	−179.8 (3)
C20—O7—C21—C26	56.3 (4)	C22—C21—C26—C25	−0.3 (4)
C20—O7—C21—C22	−129.3 (3)	O7—C21—C26—C25	173.8 (2)
C6—C1—C2—C3	0.1 (4)	C21—C26—C25—C24	−0.4 (4)
C1—C6—C5—C4	0.5 (4)	C16—C17—C18—C19	−0.4 (5)
N2—C6—C5—C4	−176.5 (3)	C14—C19—C18—C17	−1.3 (5)
C18—C19—C14—C15	2.1 (4)	N4—C19—C18—C17	179.0 (3)
N4—C19—C14—C15	−178.2 (3)	C26—C21—C22—C23	0.7 (4)
C13—C8—C9—C10	−1.7 (4)	O7—C21—C22—C23	−173.6 (2)
O3—C8—C9—C10	173.2 (2)	C8—C13—C12—C11	1.3 (4)
C19—C14—C15—C16	−1.1 (4)	C13—C12—C11—C10	−1.8 (5)
C9—C8—C13—C12	0.5 (4)	C9—C10—C11—C12	0.5 (5)
O3—C8—C13—C12	−174.2 (2)	C21—C22—C23—C24	−0.6 (5)
C8—C9—C10—C11	1.2 (4)	C26—C25—C24—C23	0.6 (5)
C6—C5—C4—C3	−0.3 (4)	C22—C23—C24—C25	−0.1 (5)

### Hydrogen-bond geometry (Å, º)

*Cg*2 is the centroid of the C8–C13 ring.

**Table:**

*D*—H···*A*	*D*—H	H···*A*	*D*···*A*	*D*—H···*A*
N2—H2···O8^i^	0.86	2.35	3.057 (2)	140
N4—H4···O4	0.86	2.05	2.906 (2)	171
C25—H25···O8^ii^	0.93	2.57	3.448 (4)	158
C14—H14···*Cg*2	0.93	2.94	3.592 (2)	128
C17—H17···*Cg*2^iii^	0.93	2.94	3.736 (3)	144

Symmetry codes: (i) *x*−1, *y*, *z*; (ii) −*x*+2, −*y*+2, −*z*+1; (iii) *x*+1, *y*, *z*.
