# Supplementary figures and images for: Crystal structure of phenyl N-(4-nitro­phen­yl)carbamate
Source: Acta Crystallogr E Crystallogr Commun. 2015 Nov 21;71(Pt 12):o969–70. doi: 10.1107/S2056989015021544 (PMC4719927; doi:10.1107/S2056989015021544)

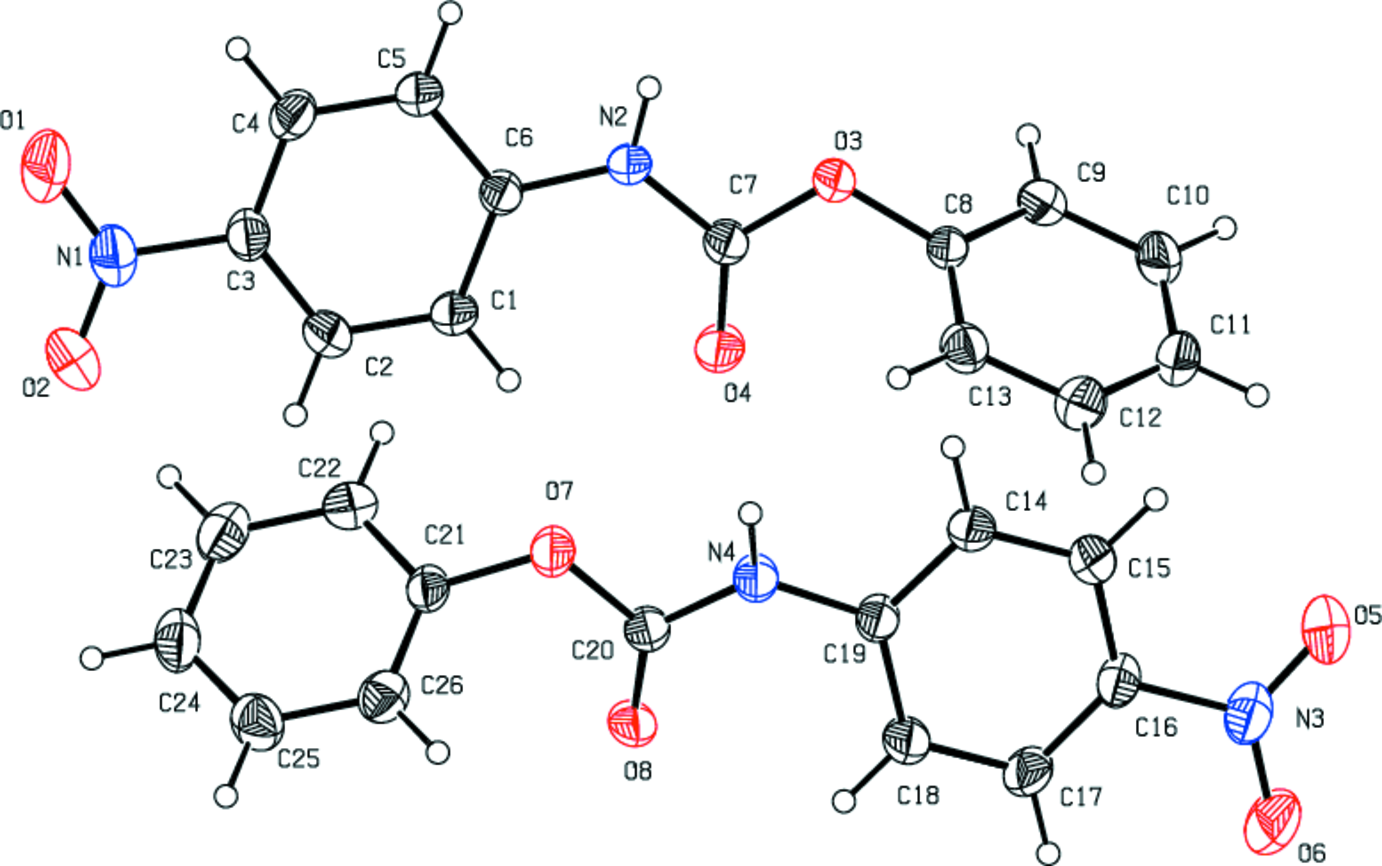

Supplement: Supplementary file 4 [file e-71-0o969-fig1.tif]

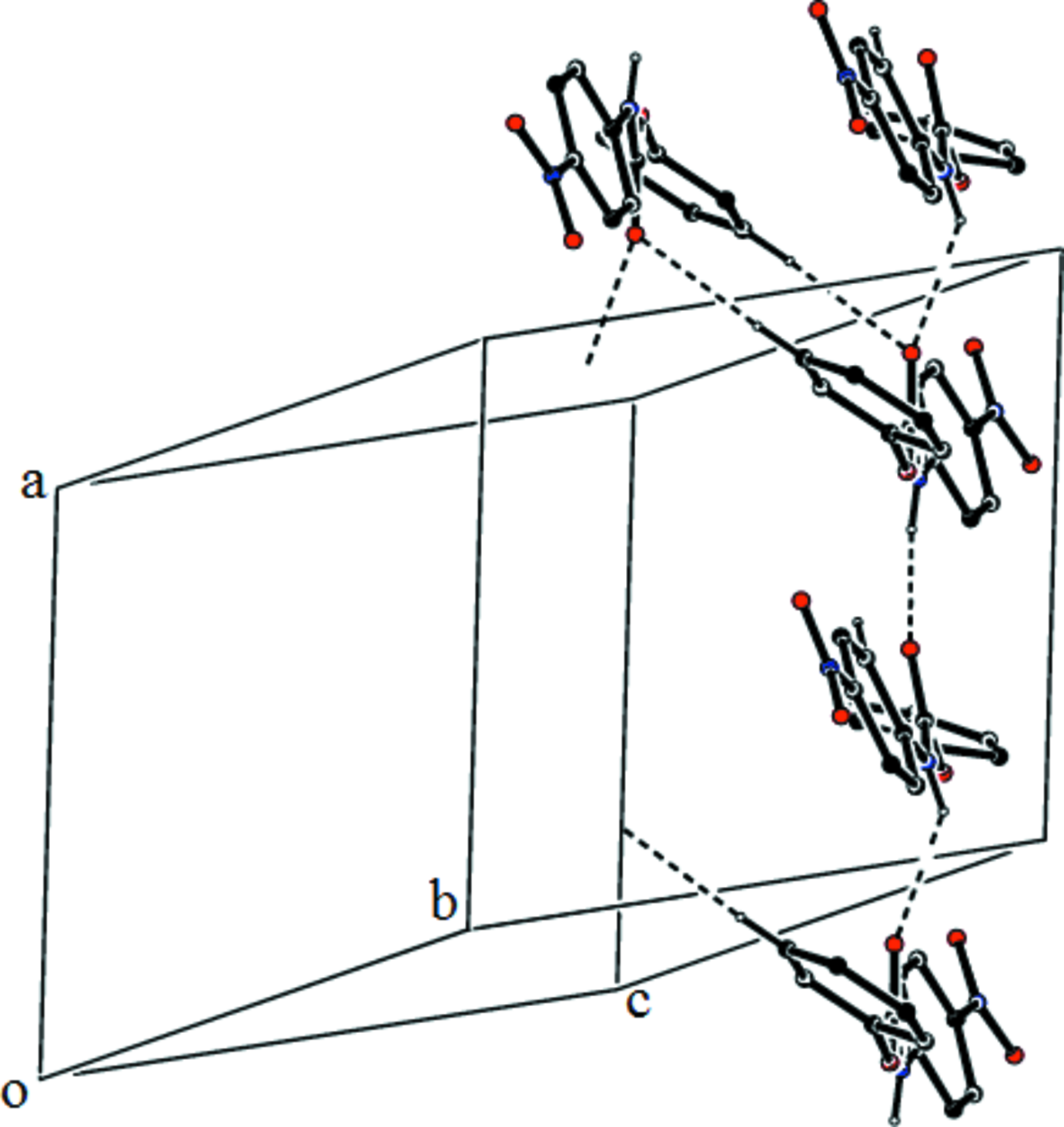

Supplement: Supplementary file 5 [file e-71-0o969-fig2.tif]
